# Overestimated SRB and Missing Girls in China

**DOI:** 10.3389/fsoc.2021.756364

**Published:** 2021-10-26

**Authors:** Li Mei, Quanbao Jiang

**Affiliations:** ^1^ School of Public Policy and Administration, Xi’an Jiaotong University, Xi’an, China; ^2^ Institute for Population and Development Studies, Xi’an Jiaotong University, Xi’an, China

**Keywords:** sex ratio at birth, missing girls, population census, overestimate, China

## Introduction

China’s SRB has been high during the past 4 decades, well exceeding the generally accepted normal range of 102–107 (United Nations Department, 1955). The SRB in China by census data was 107.63, 111.45, 119.92, and 121.21, respectively, in 1982, 1990, 2000, and 2010, and it optimistically declined to 111.30 in the just-released 2020 population census.

The high sex ratio at birth (SRB) and its related consequences like missing women and male marriage squeeze have been a hot concern in China. The Chinese government has been aware of this high SRB phenomenon and stipulated a lot of holistic regulations from prohibiting medically unnecessary abortion nationwide to cultivating a girl-friendly social context. SRB was also listed as one evaluation indicator to urge family planning officials to tackle this problem. However, past SRB and missing girls may be overestimated.

## 2020 Census Data Contradicted Past Sex Ratio at Birth Data

The 2020 census data indicated a sex ratio of 108.9 and 17.52 million more males than females for the age group 20–40. With this information, it can be calculated that in the 2020 census, men aged 20–40 numbered 214.37 million and women in this age group numbered 196.85 million. The 2020 census data quality was corroborated to be very accurate and reliable. Technically, there were a lot of improvements, including the universal adoption of computer-assisted personal interviewing devices with PADs or smartphones, the inclusion of personal ID in the questionnaire, and the real-time comparison of interviewee’s ID with that in the database. The post enumeration survey showed a 0.05 percent underreporting.

The above sex ratio and numbers, however, contradicted seriously past data officially released by the National Bureau of Statistics (NBS), as indicated in Table 1. According to annual birth numbers and SRB (or sex ratio for the population under 1 year of age in annual one in per thousand population sample survey), male births from 1980 to 2000 sums up to 239.26 million, and female births to 211.91 million, with a sex ratio of 112.91, all well exceeding the corresponding numbers in the 2020 population census. The decline in numbers can be partially attributed to deaths, but the sex ratio difference between 112.91 and 108.9 can’t simply be attributed to the difference by gender in mortality and survival levels.

**TABLE 1:** Children born between 1980 and 2000.

Year	Males (in Million)	Females (in Million)	Sex ratio (100 females)
1980	9.25	8.62	107.4
1981	10.75	10.03	107.1
1982	11.65	10.82	107.63
1983	10.72	9.94	107.9
1984	10.74	9.89	108.5
1985	11.65	10.46	111.4
1986	12.67	11.29	112.3
1987	13.22	12.07	109.6
1988	12.8	11.84	108.1
1989	12.78	11.36	112.54
1990	12.6	11.31	111.45
1991	12.28	10.37	118.33
1992	11.41	9.84	115.94
1993	11.41	9.91	115.11
1994	11.35	9.75	116.3
1995	11.1	9.53	116.57
1996	11.11	9.56	116.16
1997	10.99	9.39	117.04
1998	10.74	9.17	117.03
1999	10.39	8.7	119.35
2000	9.66	8.05	119.92
Total	239.26	211.91	112.91

## Inconsistencies of Sex Ratio by Comparison Over Time

The uncertainty of China’s SRB level was complicated by the discrepancy in data from various sources. The dataset registered by the National Family Planning Commission (merged into National Health Commission) was usually manipulated downward as the SRB was an important indicator in the family planning performance evaluation (Merli and Raftery, 2000; Zhang and Zhao, 2006). The births registered in the household registration system operated by the Ministry of Public Security (MPS) were flawed due to the severe delay in registration and due to the purposely concealments of female births (Li et al., 2010; Shi and Kennedy, 2016; Kennedy and Shi, 2019). There was also underreporting, and especially underreporting of girls in the annual one in thousand population sample survey, and population census conducted by the National Bureau of Statistics (NBS) (Zhang and Zhao, 2006; Goodkind, 2011; Cai, 2017). Besides, there were two other administrative data recently available for SRB evaluation. One is the 120 Counties Monitoring System (120 CMS), which covered 117 counties in 28 provinces, and includes a total population of 128.4 million in 2016, accounting for 9.4 percent of China’s total population. The birth information was obtained from the birth history of women, with ID checkups (Sobotka and Zhang, 2019; Jiang and Zhang, 2021). The other is the newly established Birth Registration System (BRS) in 2014 in China, which collected data from the real-name registration system for new births by health departments.

**FIGURE 1:**
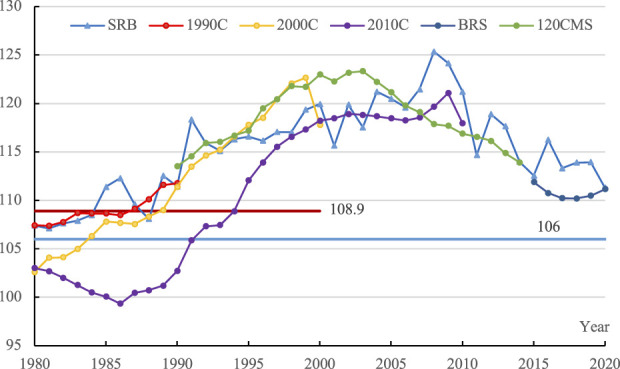
SRB by comparison over time.

The comparison of SRB showed inconsistency over the time. Comparison of the same cohort at different times is a routine procedure to discern the underreporting of female births, as concealed females would appear in later census when they needed the indispensable household registration certificate for schooling and many other aspects of social life. Below, we present the comparison of annual SRB with later data and with 120 CMS and BRS data in Figure 1.

The sex ratio for cohorts born in the 1980s and 1990s was lower in the later census, indicating the underreporting of female births and the overestimation of original SRB. As can be seen in Figure 1, the SRB for the 1980s birth cohorts intertwined the corresponding sex ratios (aged 0–10) in the 1990 census data, higher than the corresponding sex ratios (aged 10–20) in the 2000 census data, and much higher than that (aged 20–30) in the 2010 census data. Likewise, the SRB for the 1990s birth cohorts showed the same trend. Of course, we don’t mean later census data are not problematic. For example, the sex ratio for 1984–1988 cohorts was questionably lower than the normal level in the 2010 census. There was underreporting in China’s 2010 census data (Cai, 2014).

For the 120 CMS data, we can see that the curve intertwined with the 2000 census curve for the 1990s birth cohorts was higher than or intertwined with the SRB curve and was higher than the 2010 census curve for the 1990 to 2015 birth cohorts. For the 2010 population census, for people aged 5–20 (1990–2005 birth cohorts), many hidden female births were registered, namely appeared in the census. And, the birth history data from 120 CMS data may not include the out-adopted girls in the birth history. As for the BRS data for 2015 and 2020, stays at 110, equal to the 111.3 in the 2020 census, lower than the SRB obtained from the annual one in per thousand population sample survey. After the adoption of the universal two-child policy in 2016, there is little incentive to conceal births, so the BRS data should reflect the true value of the recent SRB trend.

## Underreporting of Female Births

Underreporting of female births was discerned as one contributor to high SRB, but the magnitude of its effect was controversial. Among the factors, the underreporting of female births in the form of unregistered adoption or the concealment of female births was listed as one major reason for China’s high SRB (Goodkind, 2011; Johansson and Nygren, 1991; Zeng et al., 1993; Chen et al., 2015). In the 1980s, the adoption of girls without registration accounted for half of the difference between the observed and the normal SRB (Johansson and Nygren, 1991). For the year 1989, urban SRB and rural SRB were raised by 3.6 and 4.2 for every 100 female births due to underreporting of female births (Li, 1994). Zeng et al. (1993) attributed 43 to 75 percent of the magnitude higher than the normal level to underreporting of female births. In the 1990s, at least 1/25 girls were abandoned and subsequently adopted without registration (Chen et al., 2015), which meant 4 more percentage points in the observed SRB. Notice that the 4 percentage points coincide roughly with the 4 percentage points between 112.91 and 108.90 as listed in Table 1. With China’s 2000 census data and reverse survival method, it was found that underreporting of female births contributed 5–9 percentage points to the reported SRB in the 1990s, accounting for 68–73 percent of the magnitude higher than the normal level (Chen and Zhai, 2007). Underreporting of female births contributed significantly to the rise in SRB, especially in the 1980s and 1990s, but the magnitude was controversial.

## Missing Girls Recovered

The subsequent estimates of missing girls follow China’s high SRB. With the 2000 population census, there were nominally 12.8 million, but actually, 8.5 million missing girls for cohorts born between 1980 and 2000, and the other 4.3 million were alive but hidden due to underreporting (Cai and Lavely, 2003). By the year 2010, there were over 20 million truly missing girls for cohorts born between 1980 and 2010 (Cai, 2014) (Jiang et al., 2012). However, Kennedy and Shi thought almost half of the 20 million may be due to underreporting (Kennedy and Shi, 2019).

With the newly available sex ratio of 108.9 for people aged 20–40 in China’s 2020 census, given the normal SRB of 106, there would be only 7.32 million missing girls for the 1980–2000 cohorts provided the sex ratio without intervention falls to 105 for the total age group aged 20–40 in the 2020 census, lower than past estimates.

## Conclusion

There have been claims that male births outnumbered female births up to 40 million. The normal SRB of 106 inherently means more males over females (Jiang et al., 2012), and the underreporting of female births overestimated SRB and subsequent missing girls and surplus males. The shortage of marriage-age women and the increase in involuntary bachelors (bare branches) due to high SRB may not be as pronounced as the previous studies suggest. With the universal three-child policy in a context of low fertility intention, there will be little incentive to conceal births to flaw the SRB data in the future.
